# Endocarditis in Adult Congenital Heart Disease Patients: Prevention, Recognition, and Management

**DOI:** 10.1007/s11886-024-02103-9

**Published:** 2024-08-30

**Authors:** Victoria Carvajal, Fernando Baraona Reyes, David Gonzalez, Matthew Schwartz, Angela Whitlow, Jorge R. Alegria

**Affiliations:** 1 Levine Congenital Heart Center and Sanger Heart and Vascular Institute, Wake Forest University, Atrium Health, 1001 Blythe Blvd, Suite 500, Charlotte, NC 28203 USA; 2 Department of Cardiology, Boston Adult Congenital Heart Service, Boston Children’s Hospital and Brigham and Women’s Hospital, 300 Longwood Ave, Boston, MA 02115 USA; 3 Department of Medicine, Cleveland Clinic Akron General, 1 Akron General Avenue, Akron, OH 44307 USA

**Keywords:** Infective Endocarditis, Adult Congenital Heart Disease, Infective Endocarditis in ACHD Pregnancy, Transcatheter Pulmonary Valve Infective Endocarditis

## Abstract

**Purpose of Review:**

Present an updated overview of the prevention, diagnosis, and management of infective endocarditis in adult patients with congenital heart disease.

**Recent Findings:**

Care for patients with infective endocarditis is changing in the areas of specialized teams, diagnostics, and prevention. Endocarditis teams should be involved in the care of ACHD patients. The 2023 Duke Criteria for Infective Endocarditis and the 2023 European Society of Cardiology Guidelines have updated the criteria for diagnosis including new major criteria such as CT and positron emission computed tomography with 18F-fluorodeoxyglucose (FDG) scans. Immunological, PCR, and nucleic acid-based tests are now acceptable means to isolate infective organisms. Clindamycin is no longer recommended for antibiotic prophylaxis due to resistance and side effect profile. Special considerations for antibiotic prophylaxis and management must be made for specific congenital heart diseases in adulthood and pregnant ACHD patients.

**Summary:**

Infective endocarditis (IE), a potentially devastating clinical entity, is a feared threat to the health of adults with congenital heart disease (ACHD). IE needs a systematic approach for its prevention, early diagnosis and management with a multidisciplinary IE team’s involvement. There have been changes in the diagnostics and management of IE, which is reflected in updated diagnostic criteria. Timely blood cultures and imaging continue to be the mainstay of diagnosis, however the timing of blood cultures, microbiological testing, and types of diagnostic imaging such as the positron emission computed tomography with 18F-fluorodeoxyglucose (FDG) scan are new. Bicuspid aortic valves, ventricular septal defects, transcatheter pulmonary valve replacements, and tetralogy of Fallot are diagnoses at higher risk for IE in the ACHD population. The following article will focus on the preventive strategies, in addition to novel diagnostic and therapeutic approaches of IE in ACHD patients.

**Supplementary Information:**

The online version contains supplementary material available at 10.1007/s11886-024-02103-9.

## Introduction

Infective endocarditis (IE) has seen significant changes over time. The clinical manifestations are multisystemic and are often dermatologic, neurologic, ophthalmic, cardiovascular and renal [1]. Patients with congenital heart disease (CHD) are known to be at higher risk for developing IE [2]. As this population continues to grow, it is imperative to prevent, diagnose, and manage these high-risk patients to minimize morbidity and mortality. More than half of this population is made up women; therefore, additional considerations in diagnosis and management of IE are needed in adults with congenital heart disease (ACHD) during pregnancy [3].

## Scope of the Infective Endocarditis in ACHD

The incidence of IE among ACHD was 27–44 times that reported for contemporary adults in a study conducted in the Netherlands (1.33 cases per 1000 persons per year) [2]. Not all CHD has the same risk to develop IE, and several studies have been conducted to risk-stratify the complexity of CHD lesions, other co-morbidities, subsequent adverse events, and mortality related to IE. Among varied CHD diagnoses, IE in ventricular septal defects and bicuspid aortic valves were common in a retrospective study by Mano et al. which analyzed IE cases in ACHD over several decades, mainly affecting aortic, tricuspid, and mitral valves [4]. The ESC-EORP-EURO-ENDO study, a prospective international registry of IE patients with 365 ACHD patients (11.7%) also found that IE most often affected the left-sided valves.

Higher rates of IE in ACHD have also been found after dental procedures compared to non-CHD patients (14% vs 7%), with greater number of positive blood cultures for *Streptococcus viridans* (16.4% vs 8.8%, p < 0.001), which highlights the importance of antibiotic prophylaxis in this population [5].

In-hospital mortality of IE in ACHD has been found to be lower than the general population, ranging between 4–9% [4–7]. This is thought to be related to their younger age and the decreased number of comorbidities in this population [5, 6]. Major adverse events and mortality among ACHD were associated with certain co-morbidities including arterial hypertension, heart failure, and end-organ disease (e.g., renal disease) [6]. There has also been higher risk of death among patients with fistula, cerebral embolus, renal insufficiency, *Staphylococcus aureus* as causative microorganism, and failure to proceed with surgery when clinically indicated [5, 8]. ACHD patients are at high risk of surgical intervention with mortality rates of ~ 40% spanning several large studies, and with early surgical intervention associated with better outcomes. [4, 6, 7]

Special attention should be placed on ACHD of childbearing age, as this population requires expert consultation for contraception and childbirth planning.

## Pathogenesis

The pathogenesis of IE involves a complex interplay between the host's immune response with virulence factors of the causative microorganisms and their interaction with structural anatomy, and hemodynamic cardiovascular substrate. The "big 3" pathogens, namely viridans group Streptococci, Staphylococcus aureus, and Enterococcus species, account for most IE cases in the general population. These pathogens express adhesins and surface molecules that facilitate their attachment to host proteins such as collagen, thrombospondin, laminin, fibrinogen, and fibronectin. These interactions are crucial for the initial adherence of bacteria to a site of endothelial damage and may also play a role in bacterial persistence and engulfment by the host cell [9].

## Infective endocarditis in ACHD

**Fig. 1:**
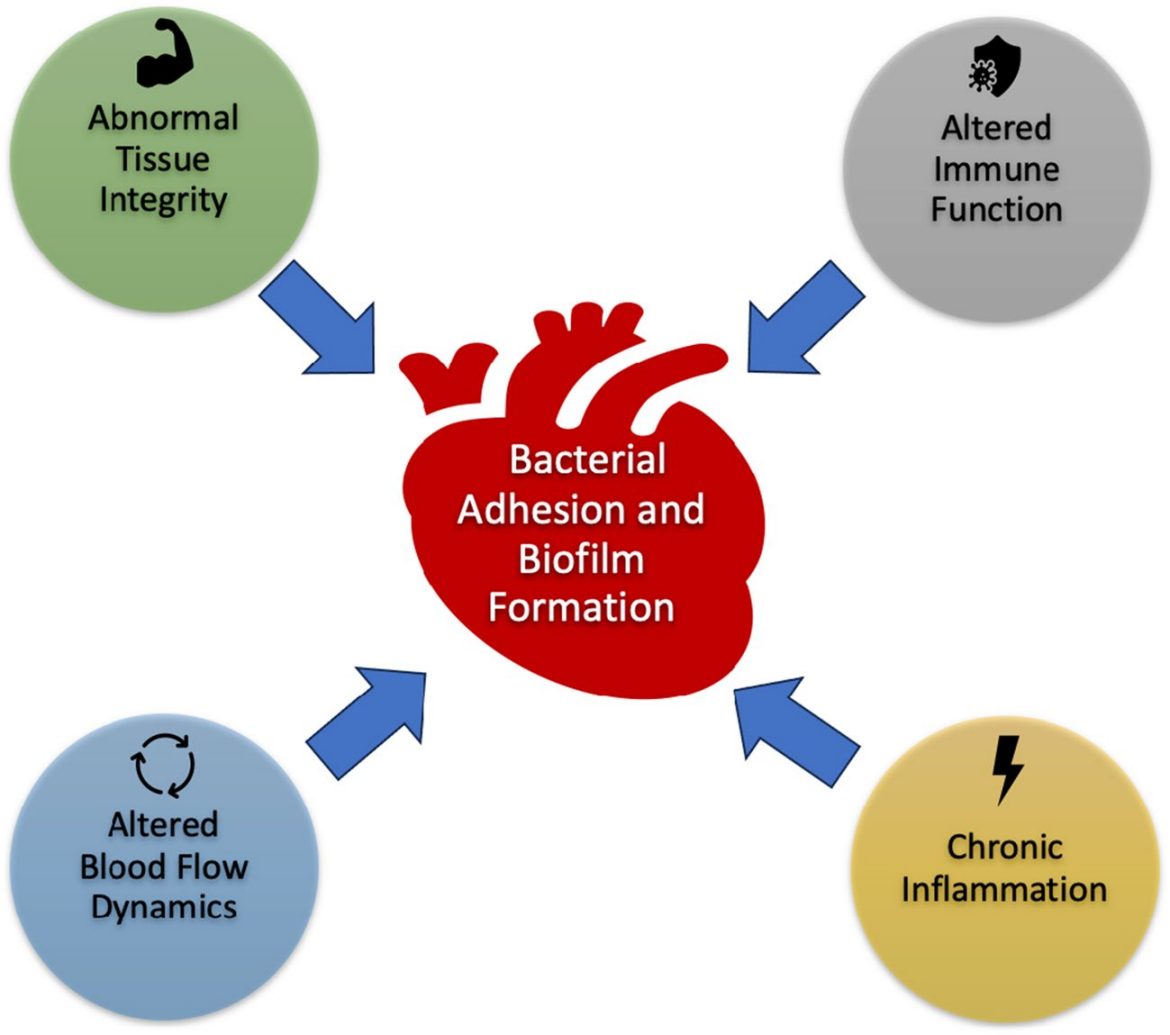
Pathogenesis of Infective Endocarditis in ACHD

The diminished host response observed in ACHD during infections can be attributed to a combination of factors related to their complex cardiovascular anomalies and altered physiological milieu (Fig. 1). Factors like chronic hypoxia, abnormal blood flow patterns stemming from their cardiac defects, valvular regurgitation, shunts, prosthetic materials including devices and valve prosthesis along with skewed white blood cell counts collectively contribute to a compromised immune system, impeding the body's ability to effectively combat infections. Chronic inflammation, a hallmark of ACHD, further exacerbates the situation.

Prolonged exposure to inflammation due to cardiac defects can eventually lead to immune system “exhaustion and dysfunction”. The altered anatomy and blood flow dynamics inherent in many congenital heart defects result in a detrimental impact. Irregular flow patterns impede immune cell function and antibody delivery to infection sites, while promoting bacterial adhesion and biofilm formation. Moreover, the compromised tissue integrity associated with ACHD can hinder the body's ability to isolate and contain infections, further impairing the host response [10].

Multi-disciplinary teams (MDTs) have shown promising outcomes and clinical benefit to patients with IE, notably improvement in short term mortality, decreased time to surgery, and an increased utilization of surgery [11]. To address the challenges and complexities of IE in ACHD, a comprehensive and multidisciplinary approach is needed. Collaborative efforts between the ACHD team, infectious disease specialists, cardiac surgeons, microbiologists, imagers, and other specialties such as neurology, nephrology, and others are essential to diagnose and treat IE and its comorbidities in ACHD effectively.

In the subpopulation of pregnant ACHD, there are specific risk factors for maternal endocarditis, such as younger age, having Medicaid coverage, and intravenous drug use. These factors should be considered along with the traditional risk factors for non-pregnant patients. Hemodynamic changes that occur during pregnancy, such as high volume and hyperdynamic state, along with relative immunosuppression, can increase the risk of endocarditis in women, particularly those with CHD. In pregnant women, symptoms of endocarditis, such as fever, systemic embolic manifestations, and new murmurs, can often be subtle. Therefore, it is essential to perform an endocarditis workup if a pregnant woman with risk factors presents with fever and other common etiologies have been ruled out [8].

## Prevention of Infective Endocarditis in ACHD

**Fig. 2:**
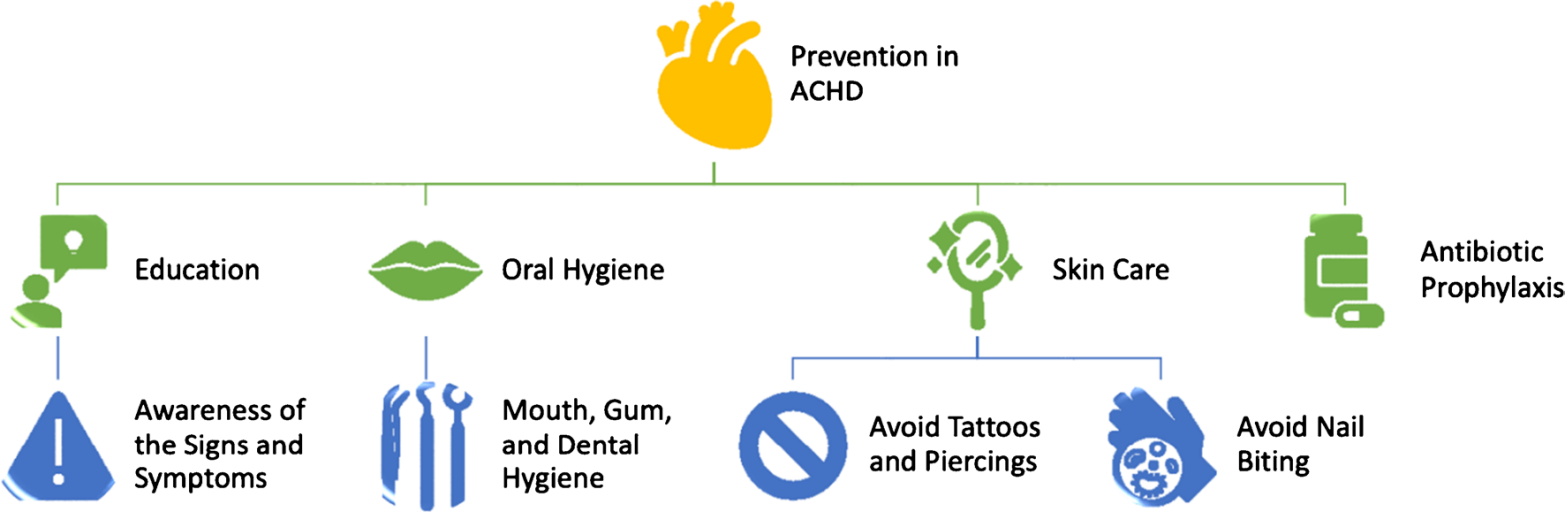
Prevention in ACHD

There are several domains that encompass preventive measures in ACHD including education, proper oral hygiene, skin care, and antibiotic prophylaxis (Fig. 2).

### Education

Providers caring for ACHD patients should continually discuss with them the potential lifelong risk of IE including its clinical presentation and importance of early diagnosis. This education should be addressed in long term follow-up and updated whenever necessary. Patients should be instructed to alert healthcare providers regarding their risk of IE and to have a low threshold to have blood cultures drawn prior to the use of antibiotics when they present with fever of unknown origin, malaise, fatigue, night sweats, arthralgias, weight loss, or other symptoms. IE may also mimic symptoms associated with neoplasia, TIA or stroke, renal dysfunction or other diseases.

Audio and visual materials may be helpful. Educational material from the Adult Congenital Heart Association and American Heart Association (AHA) regarding IE can also be provided.

### Proper oral hygiene

A significant proportion of patients with CHD are unaware of the correlation between oral health and heart disease [12]. ACHD have actually been found to have better oral health compared to heart-healthy individuals, however with higher amount of dental plaque [13]. Despite these findings, it's important to note that oral health is of particular importance in patients with CHD due to the risk of IE from oral bacteria and odontogenic oral infections [13, 14]. The American College of Cardiology (ACC) and the AHA recommend meticulous oral care and routine preventive care by a dentist or oral hygienist for patients with CHD.

### Skin Care

For patients undergoing surgical procedures involving infected skin, the European Society of Cardiology (ESC) recommends that the therapeutic regimen contain an agent active against staphylococci and beta-hemolytic streptococci [15]. The ESC also expresses concern about body piercing and susceptibility for the acquisition of IE. They recommend that these procedures should be performed under strictly sterile conditions, though antibiotic prophylaxis is not recommended [15].

Tattooing and piercing can be associated with IE, particularly in individuals with CHD who are more susceptible to this condition [16, 17]. It is recommended to avoid tattooing and piercing by the 2023 ESC Guidelines [18]. There are reported cases of IE following piercing or tattooing, with *Staphylococcus aureus* being one of the commonly isolated organisms [17]. However, the incidence of endocarditis related to tattooing appears less common compared to piercing [16].

Despite these associations, it's important to note that the role of antibiotic prophylaxis in this situation remains unclear, and active prevention of this condition requires collaboration between cardiologists, patients, and body art professionals through education and guidance [19].

Nail biting has also been anecdotally associated with IE and should also be discouraged.

### Infective Endocarditis Prophylaxis

Antibiotic prophylaxis (AP) is used to prevent IE in patients at high risk undergoing invasive dental procedures. The benefits of AP include a reduction in the incidence of IE following such procedures, particularly in high-risk individuals [20, 21]. A systematic review and meta-analysis showed that no high-risk patients developed IE across all studies where 100% of the patients were treated with prophylactic antibiotics [22]. Another study demonstrated a significant temporal association between invasive dental procedures and subsequent IE in high-IE-risk individuals, and a significant association between AP use and reduced IE incidence following these procedures [20].

The AHA and the ACC recommends AP for patients with underlying cardiac conditions associated with the highest risk of adverse outcome from IE, such as those with a prosthetic heart valve, a past history of IE, unrepaired cyanotic congenital heart disease, residual shunt adjacent to prosthetic material, and cardiac valvulopathy following cardiac transplantation [23, 24]. AP is recommended for all dental procedures that involve manipulation of either gingival tissue or the periapical region of teeth or perforation of oral mucosa in these high-risk patients [23, 24]. Antibiotic choice depends on administration route and a patient’s allergies. Notably, if there has been a reaction i.e. urticaria or anaphylaxis with a penicillin derivative, cephalosporins should not be given. 500 mg of oral azithromycin or clarithromycin are good alternatives in these cases. 100 mg of doxycycline is also acceptable. [23, 25] For optimal effect, antibiotic prophylaxis should be administered 30–60 min prior to the procedure. When oral medications can be given, 2gm of Amoxicillin is considered the gold standard. IV/IM alternatives include Ampicillin 2gm or Cefazolin 1gm (Table 1). [25]

**Table 1:** Antibiotic Regimens for a Dental Procedure Regimen: Single Dose 30 to 60 Minutes Before Procedure

Situation	Agent	Adults	Children
Oral	Amoxicillin	2 g	50 mg/kg
Unable to take oral medication	Ampicillin OR	2 g IM or IV	50 mg/kg IM or IV
	Cefazolin or ceftriaxone	1 g IM or IV	50 mg/kg IM or IV
Allergic to penicillin or ampicillin—oral	Cephalexin*†OR	2 g	50 mg/kg
	Azithromycin or clarithromycin OR	500 mg	15 mg/kg
	Doxycycline	100 mg	< 45 kg, 2.2 mg/kg > 45 kg, 100 mg
Allergic to penicillin or ampicillin and unable to take oral medication	Cefazolin or ceftriaxone†	1 g IM or IV	50 mg/kg IM or IV

Clindamycin is no longer recommended for antibiotic prophylaxis for a dental procedure

IM indicates intramuscular; and IV, intravenous

^*^Or other first- or second-generation oral cephalosporin in equivalent adult or pediatric dosing

^†^Cephalosporins should not be used in an individual with a history of anaphylaxis, angioedema, or urticarial with penicillin or ampicillin

(Reprinted with permission from Circulation ©2021;143:e963-e978 ©2021 American Heart Association, Inc.)

In addition to reducing the incidence of IE, AP is also cost-effective, particularly for high-risk patients AP has been shown to be less costly and more effective than no AP for all patients at risk of IE, with potential annual cost savings and health gains [26]. However, it's important to note that the wide-spread administration of AP can promote the emergence of resistant microorganisms, consequently reducing the efficacy and number of antibiotics available for the treatment of IE [23]. Therefore, the use of AP should be carefully considered and tailored to the individual patient's risk profile. Of note, clindamycin has been associated with severe side effects including Clostridium difficile infection, and azithromycin has been associated with prolongation of the QTc interval [27, 28]. The 2023 ESC Guidelines in IE discourages the use of clindamycin and azithromycin, while the AHA no longer recommends clindamycin due to the higher risk profile of side effects [18].

## Recognition and Diagnosis

Since Sir William Osler first described infective endocarditis in 1885 there has been an evolution in the clinical presentation, diagnosis, microbiology, and outcomes of patients with IE. In 1994, Durack and colleagues from the Duke University Medical Center proposed a diagnostic schema that stratified patients with suspected IE into definite, possible, and rejected cases [29]. This schema, known as the Duke Criteria, was based on clinical, echocardiographic, and microbiological criteria and did not rely on histopathologic confirmation of resected valvular material or arterial embolus and the Duke Criteria were modified in 2000. [29]

IE is a systemic infection that can present with a wide range of clinical manifestations, often involving multiple organ systems. The presentation can be indolent or fulminant, with symptoms including fever, anorexia, weight loss, back pain, fatigue, weakness, arthralgias, myalgias, rigors, and diaphoresis [9, 30]. Cardiac manifestations are primarily due to valvulitis, which can lead to changing cardiac auscultatory findings or the development of congestive heart failure. The most frequent complication of IE is heart failure, often resulting from destructive valve lesions and perivalvular extension of the infection [31]. Conduction abnormalities with new or progressive atrioventricular block can also be present.

Extra-cardiac manifestations can include petechiae, splenomegaly, and renal abnormalities such as glomerulonephritis or infarcts [9, 30]. Embolic events, which can affect various vascular territories, are also common and can lead to ischemic or hemorrhagic cerebral complications, splenic or renal infarctions, and septic pulmonary emboli, particularly in cases of right-sided IE [31, 32].

Isolating the infective organism and visualizing intracardiac vegetations are key in diagnosis. The Duke Criteria for Infective Endocarditis was modified in 2023 by the Duke-International Society for Cardiovascular Infectious Diseases (ISCVID) [33]. There continue to be major and minor criteria to stratify definitive, possible, and rejected IE (Table 2).

**Table 2:** Definitions of Infective Endocarditis According to the 2023 Duke-International Society for Cardiovascular Infectious Diseases Infective Endocarditis (IE) Criteria, With Proposed Changes in Bold Type

I. DEFINITE ENDOCARDITIS
A. Pathologic Criteria
(1) Microorganisms identified^a^ in the context of clinical signs of active endocarditis in a vegetation; from cardiac tissue; from an explanted prosthetic valve or sewing ring; from an ascending aortic graft (with concomitant evidence of valve involvement); from an endovascular intracardiac implantable electronic device (CIED); or from an arterial embolus
or
(2) Active endocarditis^b^ (may be acute^c^ or subacute/chronic^d^) identified in or on a vegetation; from cardiac tissue; from an explanted prosthetic valve or sewing ring; from an ascending aortic graft (with concomitant evidence of valve involvement); from a CIED; or from an arterial embolus
B. Clinical Criteria (1) 2 Major Criteria *or* (2) 1 Major Criterion and 3 Minor Criteria *or* (3) 5 Minor Criteria II. POSSIBLE ENDOCARDITIS A. 1 Major Criterion And 1 Minor Criterion *or* *B. 3 Minor Criteria* III. REJECTED ENDOCARDITIS A. Firm alternate diagnosis explaining signs/symptoms^e^ ^or^ ^B. Lack of recurrence despite antibiotic therapy for less than 4 d^ or C. No pathologic or macroscopic evidence of IE at surgery or autopsy, with antibiotic therapy for less than 4 d *or* *D. Does not meet criteria for possible IE, as above*

^a^By culture, staining, immunologic techniques, polymerase chain reaction (PCR), or other nucleic acid–based tests including amplicon (16S, 18S, internal transcribed spacers) sequencing, metagenomic (shotgun) sequencing, or in situ hybridization on fresh or paraffin-fixed tissue. Molecular techniques and tissue staining (Gram stain, periodic acid–Schiff with diastase, Grocott, or silver stains such as Warthin-Starry, Steiner, or Dieterle) should be interpreted cautiously, particularly in patients with a prior episode of IE because such tests can remain positive for extended periods following successful treatment. Antibiotic therapy before tissue procurement may also significantly alter microorganism morphology and staining characteristics. Test specificity is influenced by several factors, and false positives can occur. Test interpretation should always be in the context of clinical and histological evidence of active endocarditis. A single finding of a skin bacterium by PCR on a valve or wire without additional clinical or microbiological supporting evidence should be regarded as Minor Criterion and not Definite IE [34]

^b^Active endocarditis—vegetations, leaflet destruction, or adjacent tissue of native or prosthetic valves showing variable degrees of inflammatory cell infiltrates and healing. Many specimens demonstrate mixed features

cAcute endocarditis—vegetations or cardiac/aortic tissue lesions of native or prosthetic valves showing active inflammation without significant healing or organizational change. ^d^Subacute/chronic endocarditis—vegetations or cardiac/aortic tissue lesions of native or prosthetic valves demonstrating evidence of healing or attempted healing: maturing granulation tissue and fibrosis showing variable mononuclear cell infiltration and/or calcification. Calcification can occur rapidly in injured tissue and vegetations or be part of the underlying valvular disease that was the original nidus for IE

^e^Firm alternate diagnosis explaining IE signs and symptoms consists of either microbiologic or nonmicrobiologic causes. Firm alternate microbiologic diagnosis includes (1) identifiable source for bloodstream infection with a nontypical IE pathogen, (2) rapid resolution of bloodstream infection, and (3) absence of evidence for IE on cardiac imaging. Firm alternate nonmicrobiologic diagnosis includes (1) presence of non-IE cause for cardiac imaging findings (eg, marantic or nonbacterial thrombotic endocarditis) and (2) absence of microbiologic evidence for IE

(From: Fowler, V.G., et al., The 2023 Duke-ISCVID Criteria for Infective Endocarditis: Updating the Modified Duke Criteria. Clin Infect Dis. 2023 Aug 22;77(4):518–526. https://doi.org/10.1093/cid/ciad271, by permission of Oxford University Press) [33]

**Fig. 3:**
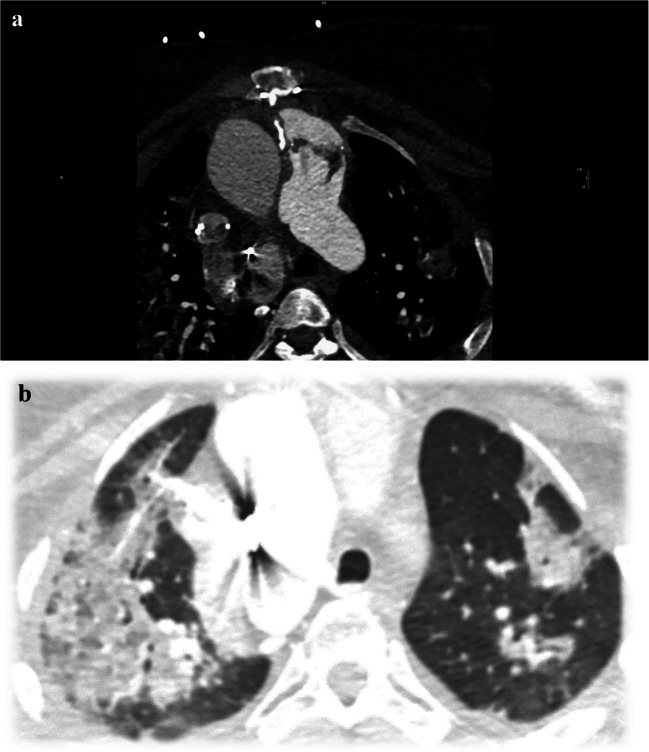
Adult patient s/p Rastelli procedure with prosthetic pulmonary valve. **A)** Cardiac CT video clip demonstrating prosthetic pulmonary valve with mobile vegetation. **B)** Chest CT with multiple septic pulmonary emboli. (Courtesy of Dr. Dan Wallihan.)

**Fig. 4:**
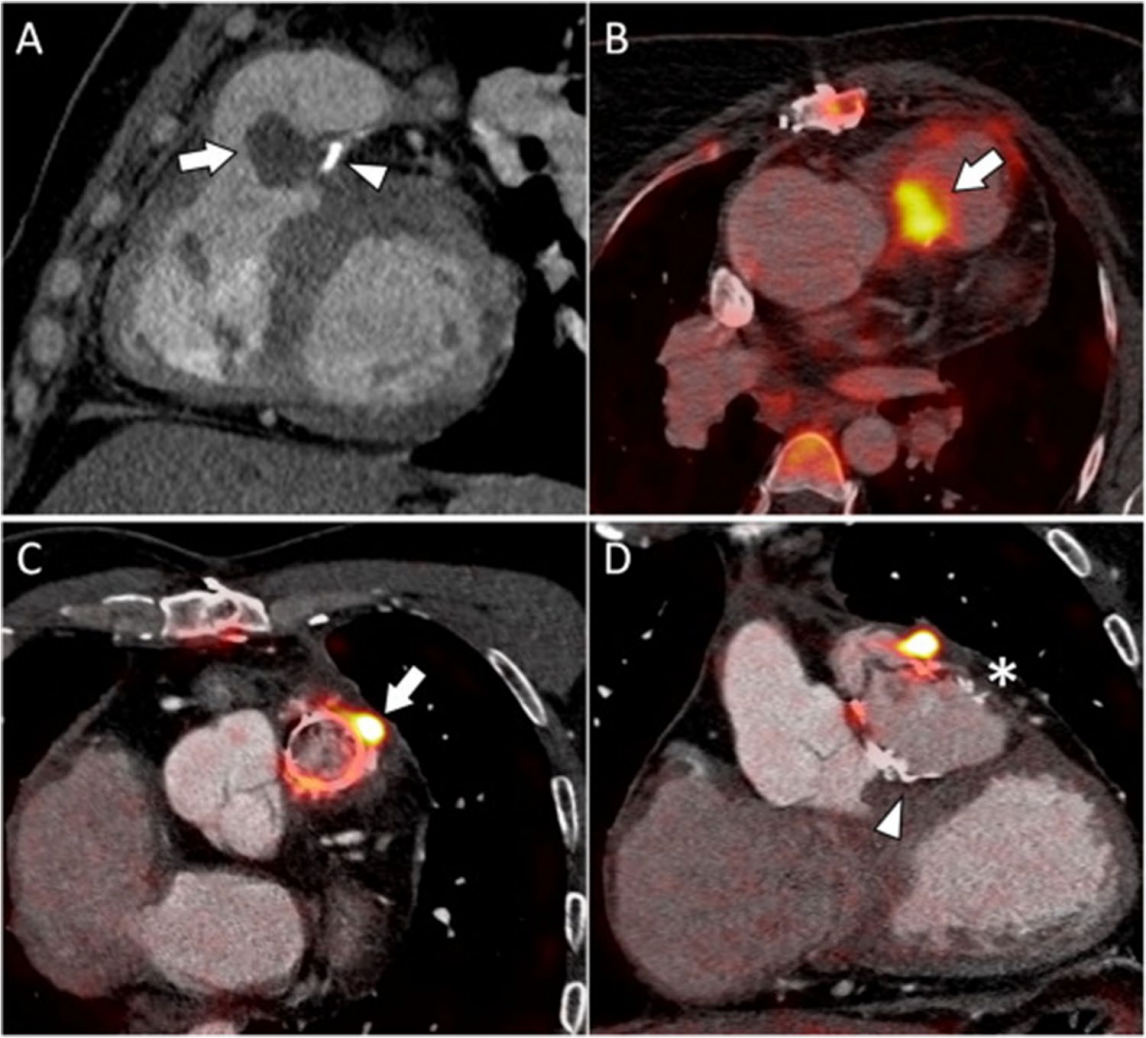
Two cases of right-sided IE confirmed by PET/CTA. A 37-year-old woman with a repaired pulmonary atresia and ventricular septal defect (VSD) (no 3). She had fever and positive blood cultures for Staphylococcus epidermidis, 1.1 years after her last surgery, which consisted on right ventricle to pulmonary artery homograft replacement. ECHO findings were inconclusive for a vegetation on the VSD patch. PET/CTA performed 3 days later showed a large (3 cm) vegetation on the pulmonary homograft (**A,** *arrow*) that was markedly hypermetabolic (SUVmax 9.93) in the fusion images, thus confirming IE (**B,** *arrow*). Another case of a 43-year-old man with tetralogy of Fallot-type CHD (no 12) who had early Staphylococcus epidermidis IE of a bioprosthetic pulmonary valve (Carpentier® Magna) 7 months after his fifth surgery. PET/CTA showed pulmonary vegetations and a periprosthetic abscess with a SUVmax of 19.28 (**C,** *arrow*) associated with septic pulmonary embolisms. He also had a right ventricular outflow tract enlargement patch of pericardium (**D,** *asterisk*), a Teflon® VSD patch (**D,** *arrowhead*), and Dacron® pulmonary artery plasties. None of them showed pathological FDG uptake or anatomical abnormalities. (Reprinted from: Pizzi, M.N., et al. Int J Cardiol, 2017. 248: p. 396–402, with permission from Elsevier) [35]

The new criteria include advancements in microbiology diagnostics, such as the use of enzyme immunoassay for Bartonella species, polymerase chain reaction, amplicon/metagenomic sequencing, and in situ hybridization. The list of "typical" microorganisms causing IE was expanded and now includes pathogens to be considered as typical only in the presence of intracardiac prostheses [33]. CT and positron emission computed tomography with 18F-fluorodeoxyglucose (FDG) are new imaging modalities that have also been used to identify intracardiac findings, particularly increasing the yield in cases with prosthetic materials (see Figs. 3 and 4). [29, 32, 35]. See Table 3 for a comparison chart of the Modified 2000 Duke Criteria and new 2023 updates.

**Table 3:** A Comparison of Revised Duke Criteria for Diagnosis of Infective Endocarditis

CRITERIA	2000	2023
PATHOLOGIC		
Microorganism Identification	Microorganisms demonstrated by culture or histologic examination of a vegetation, vegetation that has embolized, or an intracardiac abscess specimen Vegetation or intracardiac abscess confirmed by histologic examination showing active endocarditis	Microorganisms identified in appropriate sample by PCR, amplicon or metagenomic sequencing, or in situ hybridization
MAJOR CLINICAL CRITERIA Microbiology		
Blood cultures	At least 2 positive cultures of blood samples drawn > 12 h apart; or all of 3 or a majority of > 4 separate cultures of blood (with first and last sample drawn at least 1 h apart)	Removed requirements for timing and separate venipunctures for blood cultures
Definition of typical organisms	Viridans streptococci, Streptococcus bovis, HACEK group, Staphylococcus aureus; or Community-acquired enterococci, in the absence of primary focus	Added typical pathogens: 1) *S. lugdunensis; E. faecalis*; all streptococci except *S. pneumoniae* and S.pyogenes; Granulicatella spp.; Abiotrophia spp.; and Gemella spp. 2) Organisms to be considered “typical” IE pathogens in the setting of intracardiac prosthetic material: coagulase negative staphylococci, Corynebacterium striatum; C. jeikeium, Serratia marcescens, Pseudomonas aeruginosa, Cutibacterium acnes, nontuberculous mycobacteria, and Candida spp.
Other microbiologic testing	Single positive blood culture for *Coxiella burnetii* or an IgG antibody titer > 1:800	Added new Major Criteria for fastidious pathogens: 1) PCR or amplicon/metagenomic sequencing identifies C. burnetii, Bartonella spp., or T. whipplei from blood; or 2) IFA ≥ 1:800 for IgG antibodies identifies B. henselae or B. quintana
Imaging		
Echocardiography	Oscillating intracardiac mass on valve or supporting structures, in the path of regurgitant jets, or on implanted material in the absence of an alternative anatomic explanation; abscess; or new partial dehiscence of prosthetic valve	Similar to earlier versions. Cornerstone of imaging criterion
Cardiac computed tomography	N/A	Added new Major Criterion Findings equivalent to echocardiography
[18] FDG PET/CT	N/A	Added new Major Criterion Findings for native valve, cardiac device, or prosthetic valve > 3 mo after cardiac surgery are equivalent to echocardiography
Surgical	N/A	Added new Major Criterion Intraoperative inspection constitutes Major Criterion in absence of Major Criterion by cardiac imaging or histopathology
MINOR CLINICAL CRITERIA		
Predisposition	Predisposing heart condition or injection drug use	Added transcatheter valve implant/repair, endovascular CIED, and prior diagnosis of IE
Fever	> 38º C	Unchanged
Vascular phenomena	Major arterial emboli, septic pulmonary infarcts, mycotic aneurysm, intracranial hemorrhage, conjunctival hemorrhages, and Janeway's lesions	Added splenic and cerebral abscess
Immunologic Phenomena	Glomerulonephritis, Osler's nodes, Roth's spots, and rheumatoid factor	Added definition for immune complex mediated glomerulonephritis
Microbiological	Positive blood culture but does not meet a major criterion as noted above or serological evidence of active infection with organism consistent with IE	Added PCR or amplicon/metagenomic sequencing evidence of typical pathogen
Imaging	N/A	Added PET/CT evidence < 3 mo of cardiac surgery
Physical Examination	N/A	New auscultation of regurgitant murmur when echocardiography is unavailable

(From: Fowler, V.G., et al., The 2023 Duke-ISCVID Criteria for Infective Endocarditis: Updating the Modified Duke Criteria. Clin Infect Dis. 2023 Aug 22;77(4):518–526. https://doi.org/10.1093/cid/ciad271, by permission of Oxford University Press) [33]

Additional predisposing conditions were clarified, including transcatheter valve implants, endovascular cardiac implantable electronic devices, and prior IE. These updates aim to improve the diagnostic accuracy and clinical management of IE, reflecting the evolving understanding of this complex disease. The Duke-ISCVID Criteria are intended to be updated periodically and made available online as a "Living Document" [33].

Currently, there is no data available regarding the sensitivity and specificity of this new diagnostic criteria for ACHD patients, but it is foreseeable that they will help in earlier diagnosis of IE.

## Special ACHD Subpopulations and Infective Endocarditis

Not all patients with ACHD are at equal risk of developing IE. The incidence of IE has been described for certain heart lesions and many different variables including the status of repair of the heart lesion and valve replacement approaches (surgical or transcatheter) need to also be considered. These aspects will be described below. The decision for AP in these populations varies and has been debated in some cases, but further evidence may affect indications for AP in these populations. Management of IE in these cases range from medical management, interventional procedures, and surgical intervention.

### Bicuspid Aortic Valve

Patients with a bicuspid aortic valve (BAV) have a significantly higher risk of native valve IE compared to the general population, with a 12-fold increased risk and an incidence rate of 48.13 per 10,000 patients-year [36].

The clinical presentation of IE in BAV patients can be similar to patients with tricuspid aortic valves, including symptoms such as fever, chills, and a new diastolic murmur. These patients have a higher incidence of viridans group streptococci IE and IE from suspected odontogenic origin [37].

Management of IE in BAV patients often involves a combination of antibiotic therapy and, in certain cases, surgical intervention. The ACC and the AHA recommend early consultation with a cardiovascular surgeon as soon as the diagnosis of aortic valve endocarditis is made, and surgery is indicated in patients with life-threatening congestive heart failure, cardiogenic shock, annular or aortic abscesses, infections resistant to antibiotic therapy, and fungal endocarditis [38].

The use of antibiotic prophylaxis in BAV patients to prevent IE is currently a matter of debate. While it is no longer universally recommended by international guidelines, some studies suggest a potential benefit due to the high risk of IE in this population [36, 39]. However, more high-quality prospective studies are needed to accurately estimate the incidence of IE, the relative risk, and the potential benefit of antibiotic prophylaxis in BAV patients [36].

### Ventricular Septal Defect

Patients with a ventricular septal defect have an increased risk of IE compared to the general population. A study from Sweden found that the incidence of IE was 1.7–2.7 per 1000 patient-years in adults with VSDs, which is 20–30 times the risk in the general population, more so in patients with small, unrepaired VSDs [40].

A Danish population-based cohort study reported a high hazard ratio (HR) of endocarditis in patients with VSDs, both unrepaired and surgically closed. The HR of endocarditis was 28.0 in patients with unrepaired defects and 82.7 in patients with surgically closed defects [41].

However, it's important to note that these data provide ranges of risk in large populations, and the actual lifetime risk of IE in an individual patient with a VSD may be influenced by several factors, including the severity of the defect and the presence of other risk factors for IE.

### Eisenmenger Syndrome

Patients with Eisenmenger Syndrome are at significantly increased risk of IE and require antibiotic prophylaxis. Due to right to left shunt, patients can present with abscess in the brain, spleen or other systemic locations [42].

### Patent Ductus Arteriosus

Patent ductus arteriosus (PDA) can serve as a risk factor for right-sided IE [43]. This is due to the untreated left-to-right shunt that can occur in PDA, which can facilitate the adherence of bacteria to the endothelial surface, leading to the formation of vegetations characteristic of IE. The incidence of IE in patients with PDA has significantly declined over the past few decades, likely due to the widespread use of antibiotics and advancements in surgical and catheter-based closure techniques for PDA [44]. Despite this, IE can still occur in patients with PDA, especially if the ductus arteriosus remains open or recannulates after closure [45, 46]. Therefore, a high index of suspicion for IE should be maintained in patients with PDA who present with unexplained fever or other signs of infection [47].

Early diagnosis of IE in patients with PDA can be achieved through transthoracic echocardiography, CT, or PET-CT 18-FDG scan [47]. Once diagnosed, prompt antimicrobial therapy is advised to minimize the risk of complications such as multiorgan failure and severe pulmonary embolism [43].

In addition to medical management, closure of a large, hemodynamically significant PDA is recommended to reduce the risk of IE [37]. However, the routine closure of PDA solely for the prevention of IE is a matter of debate, with some studies suggesting that it may be unnecessary given the low incidence of IE in this population [44].

### Tetralogy of Fallot

Patients with tetralogy of Fallot (ToF) are considered at high risk of IE due to altered hemodynamics and multiple invasive procedures, including pulmonary valve replacement (PVR) [48]. In a study involving 1164 patients with ToF, the incidence rates of IE per 10,000 person-years were 22.4 among patients with ToF and 0.1 among controls [48]. Furthermore, PVR was associated with a further increased incidence of IE among patients with ToF, with incidence rates per 10,000 person-years of 46.7 with PVR and 2.8 without PVR [48]. Another study found that there were 393 (2.1%) endocarditis-related admissions among 18,353 admissions of adult ToF patients, and the incidence of endocarditis-related admissions increased over time [49]. In a study of 338 adult ToF patients who underwent prosthetic valve implantation, the annual incidence of prosthetic valve endocarditis was 0.4% [50]. These studies highlight the significantly increased risk of IE in patients with ToF, particularly those who have undergone PVR. Therefore, intensified awareness, preventive measures, and surveillance of this patient group are crucial.

### Fontan

IE is rare in patients with Fontan procedures [51] and there is debate if antibiotic prophylaxis is recommended [42]. The presence of cyanosis is a well known risk factor for endocarditis, and many such patients remain cyanotic for a number of different reasons.

### Following Transcatheter Pulmonary Valve Replacement

Due to the increased frequency of transcatheter pulmonary valve replacement (TPVR) in patients with congenital heart disease over the past 20 years, transcatheter pulmonary valve endocarditis requires special consideration. IE is a significant adverse event following TPVR. A multicenter study found that the cumulative incidence of IE was 9.5% at 5 years and 16.9% at 8 years post-TPVR, with an annualized incidence of 2.2 per 100 patient-years [47]. The most common causative organisms were Staphylococcus aureus and Viridans group Streptococcus species, accounting for 56% of cases [47]. Risk factors for IE included younger age, a previous history of endocarditis, and a higher residual gradient after valve implantation, but the type of transcatheter pulmonary valve did not appear to be a significant risk factor [47]. An elevated residual gradient across the valve after TPVR appears to be an especially important risk factor for subsequent endocarditis, as it has been associated with the development of endocarditis in several studies [34, 52, 53]. Furthermore, numerous studies have been published examining the risk of endocarditis after TPVR with the Melody valve, but fewer studies have examined the risk after TPVR with the Sapien valve [54–57]. A French study of 79 patients who underwent TPVR suggested a higher incidence of endocarditis after Melody valve implantation [57]. However, the large, multicenter study by McElhinney et al. evaluated over 2400 patients and showed that the incidence of endocarditis after Melody valve placement was not significantly different from the incidence after Sapien placement when other patient and procedural factors are accounted for by multivariate analysis [34].

Most patients that develop significant transcatheter valve obstruction due to endocarditis require surgical valve replacement, but some can show improvement with antibiotic therapy alone. Patients who do not develop significant obstruction and who have no clear evidence of transcatheter involvement (valvular vegetations or thickening) can often be treated with antibiotics alone [34]. In the large study by McElhinney et al., roughly 50% of patients with transcatheter pulmonary valve endocarditis were treated with antibiotics alone. [34] Antibiotic prophylaxis before indicated procedures such as dental work is important in the prevention of endocarditis after TPVR. Also, chronic anti-platelet therapy is believed to lower the risk of transcatheter pulmonary valve endocarditis. The prevention of small valve thrombi could theoretically lower the endocarditis risk by eliminating a nidus for bacterial adherence and growth. One prior study showed that abrupt discontinuation of antiplatelet therapy was a risk factor for Melody valve endocarditis [55]. The prescription of antiplatelet therapy after TPVR is ubiquitous, but further study is required to understand its true protective benefit.

### Women with CHD: Pregnancy and Contraception

For women with CHD, pre-pregnancy counseling and reproductive planning must take place with an ACHD cardiologist and a Maternal Fetal Medicine specialist familiar with the latest information on birth control, pregnancy, and CHD. Contraception is an important topic to address periodically throughout the reproductive life of a woman with CHD. The optimal contraceptive method is often chosen by effectiveness, potential side effects, compliance, and risk [58].

Endocarditis is a rare condition during pregnancy, with an incidence of 1 in 100,000 [59]. The diagnosis of IE can be difficult due to the lack of obvious clinical symptoms, especially when it involves the right-sided cardiac chambers. However, it poses a significant risk for morbidity and mortality for both mother and fetus [8]. In fact, endocarditis can occur in 0.5% of pregnant women with CHD [60].

Per the AHA, there is no indication for AP for invasive non-dental procedures, including delivery and IUD insertion. The use of AP during delivery is a topic of debate, and it is no longer recommended for vaginal or cesarean delivery [60]. The ACC/AHA Guideline suggest considering antibiotic prophylaxis against IE before vaginal delivery at the time of membrane rupture in select patients with the highest risk of adverse outcomes [61].

Isolating the pathogen in question and imaging continue to be key in diagnosis. It is important to consider ideal imaging modalities for fetal safety. Echocardiograms (transthoracic and transesophageal) are safe for the fetus and are the initial step in the diagnostic process. Magnetic resonance imaging (MRI) without gadolinium contrast is also safe and may help to identify complications such as aortic root pseudoaneurysms, sinus of Valsalva aneurysms, and embolic vascular lesions [62]. If other modalities are unavailable or do not provide a definitive diagnosis, the risks and benefits of computed tomography (CT) with contrast should be considered.

The management of endocarditis in pregnant patients is similar to that of non-pregnant patients. However, it is vital to ensure the safety of antibiotic use to prevent fetal toxicity. During all trimesters of pregnancy, penicillin, ampicillin, amoxicillin, daptomycin, erythromycin, mezlocillin, oxacillin, and cephalosporins are generally considered safe. Aminoglycosides and tetracyclines, on the other hand, should be avoided. [60]

### Cardiac Implantable Electronic Devices

Cardiac implantable electronic devices (CIEDs), such as pacemakers and implantable cardioverter-defibrillators, have been associated with an increased risk of IE [63]. The incidence of CIED-related endocarditis has been rising, likely due to the increased use of these devices and the aging of the recipient population. [64]

Risk factors for CIED infections include diabetes mellitus, heart failure, renal dysfunction, oral anticoagulant use, long-term corticosteroid use, and the presence of more than 2 pacing leads [64]. Procedural characteristics, such as fever within 24 h after implantation, use of preprocedural temporary pacing, and early reintervention, have also been associated with an increased risk of CIED infection [64]. In a CIED-associated infection, complete device removal and antimicrobial therapy are recommended [65]. Staphylococcus aureus is the most detected microorganism in CIED-related endocarditis.

Early diagnosis of CIED-related endocarditis is crucial for appropriate management. The use of 18F-fluoro-2-deoxyglucose (FDG) positron emission tomography/computed tomography (PET/CT) has also been suggested to improve the early diagnosis of endocarditis and infection of the implantable device [66].

## Medical Therapy

The principles of medical therapy in IE are centered around the eradication of the causative organism, typically using bactericidal antimicrobial agents. The AHA and the ESC provide comprehensive guidelines on the antibiotic management of IE [15, 32].

The first step in the treatment of IE is the identification of the causative organism, followed by in vitro determination of its susceptibility to antimicrobial agents [6]. The choice of treatment is then guided by these findings, with a preference for bactericidal over bacteriostatic agents [2, 3]. The AHA and ESC recommend the use of a combination of antimicrobials with synergistic activity, typically a cell-wall-active agent and an aminoglycoside [15, 32].

The mode of administration of these agents is also important. Treatment should be instituted parenterally to ensure complete bioavailability, high serum concentrations, and good penetration into the vegetations [6]. The timing of administration should be such that agents are given at the same time or close together to maximize the synergistic killing effect on the infecting pathogen [32]. The duration of therapy is typically 4–6 weeks, sufficient to prevent failure or relapse [3, 67]. The AHA recommends that the counting of days for the duration of therapy begin on the first day on which blood cultures are negative in cases where blood cultures were initially positive [32].

Monitoring of therapy is crucial, and while the clinical relevance of serum bactericidal titer is poor due to lack of standardization and its poor predictive value of failure, it is still considered part of the rigorous monitoring process [67].

## Cardiac Surgery

Cardiac surgery plays a fundamental role in the management of IE, particularly in cases where antibiotic treatment alone is unlikely to be curative or may be associated with ongoing risk of complications [68]. The primary objectives of surgery are the total removal of infected tissues and reconstruction of cardiac morphology, including repair or replacement of the affected valve(s) [15]. The ESC and the European Association for Cardio-Thoracic Surgery (EACTS) recommend that valve repair is favored whenever possible, particularly when IE affects the mitral or tricuspid valve without significant destruction [15].

The ACC and the AHA Guidelines indicate surgery for patients with life-threatening congestive heart failure or cardiogenic shock due to surgically treatable valvular heart disease with or without proven IE [24]. Surgery is also recommended in patients with annular or aortic abscesses, heart block, recurrent emboli on appropriate antibiotic therapy, infections resistant to antibiotic therapy, and fungal endocarditis [15, 24]. Even in complex IE cases during pregnancy, cardiac surgery may be necessary. Although feasible during pregnancy, it can have significant maternal and fetal morbidity [69]. Therefore, it should only be considered in specific cases and performed by experienced teams.

The Infectious Diseases Society of America (IDSA) and the AHA also suggest surgical intervention for fungal IE, infection with aggressive antibiotic-resistant bacteria, left-sided IE caused by gram-negative bacteria, persistent infection with positive blood cultures after 1 week of antibiotic therapy, or one or more embolic events during the first 2 weeks of antimicrobial therapy [29].

The timing of surgery in IE has been less robustly studied and predominantly based on expert consensus [68]. It has been described that early surgery in patients with IE and large vegetations significantly reduced the composite endpoint of death from any cause and embolic events by effectively decreasing the risk of systemic embolism [70]. The approach to cardiac surgery in patients with IE and a history of stroke or transient ischemic attack (TIA) is complex and requires a multidisciplinary team approach with careful consideration of the timing of surgery and the potential risks and benefits. According to the AHA, studies have suggested better outcomes for IE patients with ischemic stroke who undergo early cardiac surgery. The latency between the neurological event and cardiac surgery was not found to be a significant factor with respect to the perioperative neurological complication rate or the postoperative neurological recovery rate. Early surgery was associated with a numerically but not significantly higher hospital mortality rate after adjustment for other risk factors [29]. The American Association for Thoracic Surgery (AATS) advises that anticoagulation increases the risk of hemorrhagic conversion of an ischemic stroke and brain hemorrhage. Therefore, the team must decide whether anticoagulation is necessary, what to use, and what international normalized ratio or partial thromboplastin time level to target [71].

The ESC recommends early consultation with a cardiac surgeon to determine the best therapeutic approach. Each case must be individualized, and all factors associated with increased risk identified at the time of diagnosis [15]. A systematic review and meta-analysis found that cardiac surgery for IE within 30 days of intracranial hemorrhage was not associated with higher mortality, but did increase the rate of neurological deterioration [11, 63, 64]. The decision to proceed with cardiac surgery in patients with IE and a history of stroke or TIA should be individualized, considering the timing of the neurological event, the risk of perioperative complications, and the potential benefits of surgery.

## Conclusions

IE presents a complex and multifaceted clinical challenge for ACHD. This population's unique anatomical and physiological characteristics, as well as their prior surgical, percutaneous interventions, cardiac implantable devices, render them susceptible to IE. As such, a comprehensive approach to IE prevention, including comprehensive education and appropriate antibiotic prophylaxis, and patient education, is necessary in the care of ACHD patients. Early recognition and prompt treatment of IE are paramount to reduce the associated morbidity and mortality. A collaborative effort between ACHD cardiologists, ACHD surgeons, imagers and infectious disease experts is needed to tailor interventions to individual patient needs and optimize outcomes. Continued research into preventive strategies, risk stratification, and novel diagnostic and therapeutic options are essential to further improve the quality of life for ACHD patients at risk of IE.

## Supplementary Information

Below is the link to the electronic supplementary material.

Supplementary file1 (MP4 414 KB)
