# Correction to: Endocarditis in Adult Congenital Heart Disease Patients: Prevention, Recognition, and Management

**DOI:** 10.1007/s11886-025-02268-x

**Published:** 2025-07-22

**Authors:** Victoria Carvajal, Fernando Baraona Reyes, David Gonzalez, Matthew Schwartz, Angela Whitlow, Jorge R. Alegria

**Affiliations:** 1 Levine Congenital Heart Center and Sanger Heart and Vascular Institute, Wake Forest University, Atrium Health, 1001 Blythe Blvd, Suite 500, Charlotte, NC 28203 USA; 2 Department of Cardiology, Boston Adult Congenital Heart Service, Boston Children’s Hospital and Brigham and Women’s Hospital, 300 Longwood Ave, Boston, MA 02115 USA; 3 Department of Medicine, Cleveland Clinic Akron General, 1 Akron General Avenue, Akron, OH 44307 USA

**Correction to: Current Cardiology Reports (2024) 26:1031–1045**

10.1007/s11886-024-02103-9

The article “Endocarditis in Adult Congenital Heart Disease Patients: Prevention, Recognition, and Management”, was originally published electronically on the publisher’s internet portal on 30 August 2024 with error in the 5th author’s surname. ‘Whiltlow’ should be corrected to ‘Whitlow’.

The original article has been corrected.

